# Ultrasensitive hybridization capture: Reliable detection of <1 copy/mL short cell-free DNA from large-volume urine samples

**DOI:** 10.1371/journal.pone.0247851

**Published:** 2021-02-26

**Authors:** Amy Oreskovic, Barry R. Lutz

**Affiliations:** Department of Bioengineering, University of Washington, Seattle, Washington, United States of America; Defense Threat Reduction Agency, UNITED STATES

## Abstract

Urine cell-free DNA (cfDNA) is a valuable non-invasive biomarker with broad potential clinical applications, but there is no consensus on its optimal pre-analytical methodology, including the DNA extraction step. Due to its short length (majority of fragments <100 bp) and low concentration (ng/mL), urine cfDNA is not efficiently recovered by conventional silica-based extraction methods. To maximize sensitivity of urine cfDNA assays, we developed an ultrasensitive hybridization method that uses sequence-specific oligonucleotide capture probes immobilized on magnetic beads to improve extraction of short cfDNA from large-volume urine samples. Our hybridization method recovers near 100% (95% CI: 82.6–117.6%) of target-specific DNA from 10 mL urine, independent of fragment length (25–150 bp), and has a limit of detection of ≤5 copies of double-stranded DNA (0.5 copies/mL). Pairing hybridization with an ultrashort qPCR design, we can efficiently capture and amplify fragments as short as 25 bp. Our method enables amplification of cfDNA from 10 mL urine in a single qPCR well, tolerates variation in sample composition, and effectively removes non-target DNA. Our hybridization protocol improves upon both existing silica-based urine cfDNA extraction methods and previous hybridization-based sample preparation protocols. Two key innovations contribute to the strong performance of our method: a two-probe system enabling recovery of both strands of double-stranded DNA and dual biotinylated capture probes, which ensure consistent, high recovery by facilitating optimal probe density on the bead surface, improving thermostability of the probe-bead linkage, and eliminating interference by endogenous biotin. We originally designed the hybridization method for tuberculosis diagnosis from urine cfDNA, but expect that it will be versatile across urine cfDNA targets, and may be useful for other cfDNA sample types and applications beyond cfDNA. To make our hybridization method accessible to new users, we present a detailed protocol and straightforward guidelines for designing new capture probes.

## Introduction

Urine cell-free DNA (cfDNA) is generated from transrenal excretion of circulating cfDNA, as well as from local degradation of cells shed along the urogenital tract [1]. It is a versatile noninvasive biomarker with applications in cancer detection [1–3], infectious disease diagnosis [4,5], organ transplant monitoring [6,7], and prenatal genetic screening [1,8]. To ensure reliable outcomes of urine cfDNA assays, optimal pre-analytical methodology is essential. Several studies have compared collection procedures, storage conditions, and sample preparation methods for urine cfDNA [9–13], but there remains no consensus on best practices, and the conclusions for more well-studied plasma cfDNA may not apply to urine cfDNA. Here, we aimed to address deficiencies in one of many critical pre-analytical variables that may impact the performance of urine cfDNA tests: the DNA extraction step.

Conventional silica-based DNA extraction methods are not suitable for urine cfDNA, which is dilute and extensively fragmented. While plasma cfDNA has a peak fragment length of approximately 167 bp, reflecting protection of histone-associated DNA within nucleosomes [14], urine cfDNA is more fragmented due to glomerular filtration of the transrenal fraction and fast degradation kinetics of all cfDNA in urine. The distribution of fragment lengths varies across samples, but the majority of urine cfDNA fragments are expected to be <100 bp [8,15–17], with a wider distribution of fragments around the peak fragment length compared to plasma cfDNA [16]. Some forms of urine cfDNA, including fetal [8], tumor [18], mitochondrial [8,15], microbial [15], and viral [15] cfDNA, are even more fragmented than human nuclear cfDNA. For these particularly short forms of urine cfDNA, the peak fragment length may be as short as 30 to 60 bp [8,15]. Both DNA extraction and sequencing library preparation methods may underestimate the prevalence of short fragments, making it difficult to determine the true fragment length distribution of urine cfDNA. Recently, new single-stranded library preparation methods for next-generation sequencing have revealed that very short, formerly undetectable fragments make up a larger fraction of both plasma [14,19,20] and urine [15] cfDNA than previously recognized. Although thorough characterization of the full diversity of urine cfDNA using extraction and library preparation methods sensitive to the shortest fragments remains incomplete, it is increasingly clear that targeting short fragments is critical to maximizing the sensitivity of urine cfDNA assays.

As further evidence of the importance of targeting short fragments, decreasing PCR amplicon length has been shown to increase both clinical sensitivity and the detected concentration of urine cfDNA [9,21,22]. Detection sensitivity for fetal cfDNA in maternal urine was 25%, 75%, and 100% for PCR amplicon lengths of 65 bp, 39 bp, and 25 bp, respectively [9]. The sensitivity of Epstein-Barr virus detection similarly increased from 28% to 56% as the amplicon length decreased from 76 bp to 59 bp, and was accompanied by a 24-fold increase in median detected urine cfDNA concentration [21]. In another study, a modest 10 bp decrease in PCR amplicon length from 49 bp to 39 bp resulted in more than 10-fold increase in the detected concentration of tuberculosis (TB) cfDNA in urine [22].

The ability to detect short fragments is particularly important given the low total concentration of cfDNA in urine (<1–200 ng/mL) [1,10,23] and potential for very low copy numbers of target-specific cfDNA. To ensure maximum sensitivity for detection of low-concentration, fragmented cfDNA, it is critical not only to amplify short targets, but also to use a DNA extraction method capable of retaining short fragments with high efficiency. Conventional DNA extraction methods based on adsorption of DNA to silica under chaotropic conditions [24] are inadequate for urine cfDNA because recovery decreases with decreasing fragment length and concentration. The driving forces of DNA adsorption to silica—namely hydrophobic interactions as a result of dehydration of silica and DNA surfaces and hydrogen bonding between silica and the DNA backbone—are proportional to DNA fragment length [25]. The lower length limit of silica-based methods varies across binding matrices and buffer conditions, but recovery generally decreases for fragments less than 50–100 bp. Silica-based methods may also perform poorly for dilute samples like urine cfDNA because a non-trivial fraction of DNA may remain irretrievably bound to the silica surface [26,27]. We previously compared several silica-based methods for urine cfDNA extraction, both commercially-available kits and lab-based protocols, and discovered that none maintained high recovery across all fragment lengths from 25–150 bp [28]. Many had low or undetectable recovery of 25–40 bp fragments [28], which are expected to make up a significant fraction of urine cfDNA. Even if all other pre-analytical variables are optimized, low recovery of short fragments during the DNA extraction step risks compromising clinical results.

**Fig 1:**
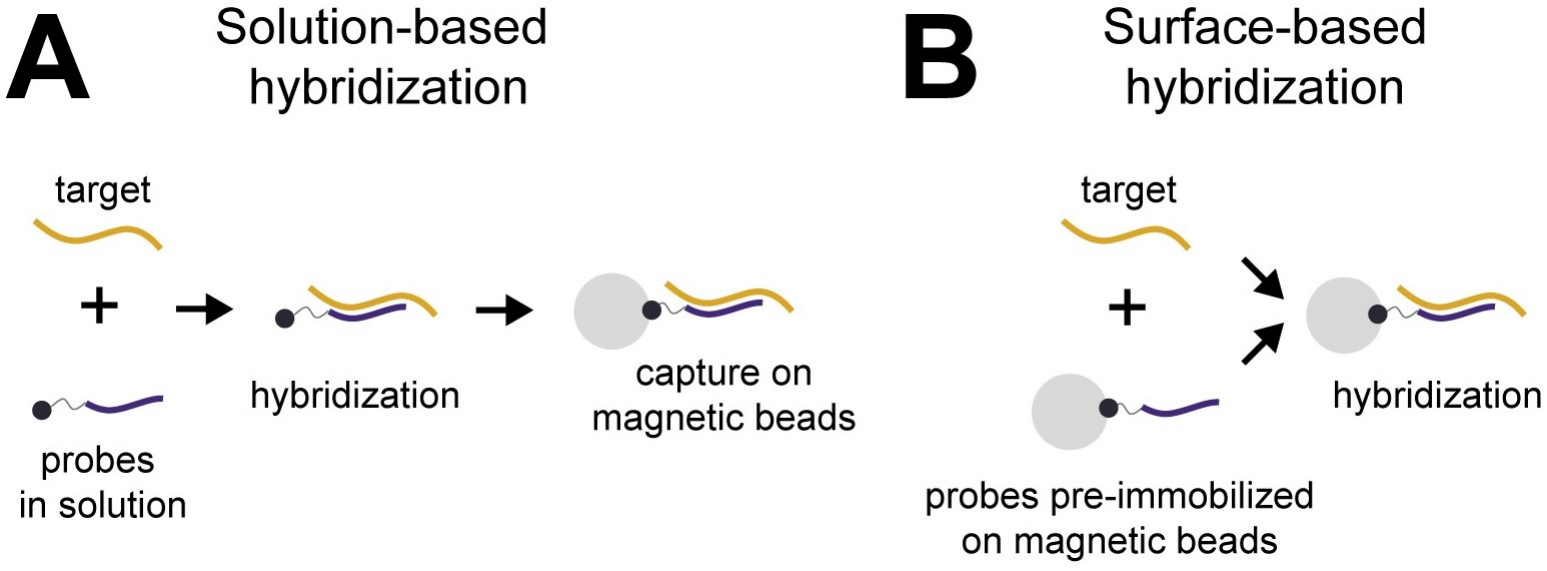
Solution-based versus surface-based hybridization capture. **(A)** Solution-based hybridization hybridizes probes to target in solution, followed by capture of probe-target complexes on magnetic beads. **(B)** Surface-based hybridization hybridizes target to probes that are pre-immobilized on magnetic beads.

We aimed to develop an improved method for extraction of short cfDNA from urine. Our goal was to improve purification and detection of known target sequences for diagnostic applications, rather than to extract total urine cfDNA. We identified sequence-specific hybridization capture as a technique likely to perform well for short, dilute urine cfDNA because it should be agnostic to DNA fragment length and concentration. Sequence-specific purification uses oligonucleotide probes that are complementary to the sequence(s) of interest to capture target-specific nucleic acids via hybridization. The capture probes are immobilized on a substrate (commonly magnetic beads) to allow for isolation and concentration of the target-specific nucleic acid. Generally, there are two hybridization capture formats distinguished by the order of the target-probe hybridization and probe immobilization steps. The first, referred to here as solution-based hybridization, involves hybridization of biotinylated probes to target in solution, followed by capture of target-probe duplexes on streptavidin-coated magnetic beads (Fig 1A). The second, referred to here as surface-based hybridization, pre-immobilizes probes on magnetic beads via biotin-streptavidin binding or covalent coupling. The probe-bead complexes are then added to the sample, where target hybridizes directly to the immobilized probes (Fig 1B).

Hybridization capture has been previously used for sequence-specific purification of nucleic acids directly from a variety of sample types, as summarized in Table 1. In these studies, hybridization capture was used as the primary sample preparation method to enrich target nucleic acid directly from raw samples or crude lysates with minimal processing (such as lysis and/or centrifugation, but no precipitation or column purification). We excluded studies that enriched bacterial samples by culture [29–32], pre-purified nucleic acids [33–39], or pre-amplified nucleic acids before hybridization (*e*.*g*., targeted enrichment of sequencing libraries). From this survey of previous applications of hybridization capture for sample preparation, the versatility and potential high sensitivity and specificity of the technique are clear. The best reported analytical sensitivities are in the range of 5–100 copies, genome equivalents, or colony forming units, with a maximum sample input of 1 mL. Purification efficiencies, when reported, were variable (<10% to 97%) but suggest the potential for high recovery if conditions are optimized. Unfortunately, many previous studies did not report detailed protocols or characterization of their methods (limit of detection, percent recovery, etc.), making direct comparison difficult. Non-optimal probe concentrations, bead volumes, and probe:bead ratios may have resulted in differences in performance across protocols. Reports on the performance of solution-based versus surface-based hybridization are inconsistent, with no consensus on when one approach may be preferred to the other.

**Table 1:** Previous uses of sequence-specific hybridization capture for sample preparation without bacterial culture, pre-purification, or pre-amplification.

Study	Hybridization format^a^	Target	Sample type(s)	Sample input volume^b^	Percent recovery	Analytical sensitivity
**Albretsen *et al*. (1990)** [40]	Surface	Measles morbillivirus RNA	Crude cell lysate	Not reported	40–45%	630 amol (3.8 x 10^8^ copies)
**Muir *et al*. (1993)** [41]	Surface	Enterovirus RNA	CSF, stool, saliva, blood, pericardial fluid, urine, solid tissue	100 μL	Not reported	Not reported
**Heermann *et al*. (1994)** [42]	Solution	Hepatitis B DNA	Serum	50 μL	Not reported	10–100 GE/mL
**Jacobsen (1995)** [43]	Surface	*Psuedomonas fluorescens* DNA	Rhizosphere soil	0.5 mg	Not reported	50 CFU
**Mangiapan *et al*. (1996)** [44]	Solution	*Mycobacterium tuberculosis* DNA	Pleural fluid	550 μL	Not reported	5–10 GE
**Beaulieux *et al*. (1997)** [45]	Surface	Enterovirus RNA	Virus supernatant	100 μL	Not reported	300 GE/mL
**Brugiere *et al*. (1997)** [46]	Solution	*Mycobacterium tuberculosis* DNA	Bronchoalveolar lavage fluid	550 μL	Not reported	Not reported
**Arnal *et al*. (1999)** [47]	Surface	Hepatitis A RNA	Stool and shellfish	200 μL	Not reported	Not reported
**Amagliani *et al*. (2006)** [48]	Surface	*Listeria monocytogenes* DNA	Milk	Not reported	Not reported	10 CFU/mL
**Thompson *et al*. (2006)** [49]	Solution	*Salmonella* DNA	Water	20 μL	47%	5 cells
**Yeung *et al*. (2006)** [50]	Solution and surface (separately; microfluidic device)	*Escherichia coli* DNA	Cell lysates	2 μL	75% (solution); 30% (surface)	100–1000 cells/mL (solution)
**Parham *et al*. (2007)** [51]	Surface	Group B Streptococci DNA	Vaginal and anal swabs	<30 μL	<10% (≥10,000 copies)– 60% (100 copies)	1250 CFU/mL
**Vansnick *et al*. (2007)** [52]	Solution	*Mycobacterium avium* subspecies *paratuberculosis* DNA	Stool and tissue	500 μL	Not reported	100 bacilli
**Peeters *et al*. (2012)** [53]	Solution and surface (separately)	Human papillomavirus DNA	Vaginal swabs	Not specified (<200 μL)	4% (solution), 25% (surface)	Not reported
**Rodriguez *et al*. (2012)** [54]	Solution	*Batrachochytrium dendrobatidis* DNA	Amphibian skin swabs	10 μL	Not reported	Not reported
**Adams *et al*. (2015)** [55]	Surface	Respiratory syncytial virus RNA	N/A	25 μL	20 (30 min)– 77% (3 hr)	Not reported
**Rohrman *et al*. (2015)** [56]	Surface (lateral flow)	Human immunodeficiency virus DNA	Blood	20 μL	Not reported	10,000 copies
**Guo *et al*. (2015)** [57]	Surface	Hepatitis B DNA	Serum	10 μL	74% (100 copies)– 97% (10^6^ copies)	90 IU/mL
**Reed *et al*. (2016)** [58]	Solution	*Mycobacterium tuberculosis* DNA	Sputum	950 μL	Not reported	20 CFU/mL (340 copies/mL)
**Reed *et al*. (2017)** [59]	Solution	*Mycobacterium tuberculosis* DNA	Sputum	950 μL	Not reported	5 CFU/mL (85 copies/mL)
**Oreskovic *et al*. (2019)** [28]	Solution	*Mycobacterium tuberculosis* DNA	Urine	1000 μL	73–84%	10 copies/mL

GE = genome equivalents; CFU = colony forming units; IU = international unit.

^a^ All studies used magnetic beads unless otherwise noted.

^b^ Sample input volume is given as volume after any concentration or centrifugation steps, if relevant.

In previous implementations, hybridization capture was used primarily to isolate target DNA from an excess of non-target DNA, which can inhibit downstream amplification and reduce sensitivity, particularly for dilute targets in an excess of non-target DNA [44,56,58]. For this reason, hybridization has been commonly used for environmental samples and pathogen detection in samples with large amounts of human gDNA (eg, stool, sputum, whole blood). For urine cfDNA, however, the total nucleic acid concentration is low and unlikely to inhibit downstream amplification. In this case, we instead aimed to leverage hybridization’s ability to sensitively capture short fragments regardless of length and concentration. To enable detection of very low target concentrations, we had to push the limits of hybridization capture to further improve the analytical sensitivity and to increase the input sample volume.

Here, we report the development and optimization of an ultrasensitive hybridization method that uses dual biotinylated sequence-specific probes immobilized on streptavidin-coated magnetic beads to capture, concentrate, and purify target-specific cfDNA fragments from 10 mL urine samples. Our hybridization method is intended for sample preparation prior to qPCR-based detection of known target sequences, such as in the case of pathogen detection; it is not suitable for applications requiring purification of total cfDNA prior to sequencing. We have identified key parameters affecting hybridization efficiency and implemented designs to ensure consistent, high percent recovery. We report full characterization of the analytical performance of our hybridization capture method and describe two key innovations that contribute to its unprecedented sensitivity: dual biotinylated capture probes and a two-probe system for recovery of both strands of double-stranded DNA (dsDNA). Our hybridization method improves upon the analytical performance of both existing silica-based urine cfDNA extraction methods and hybridization capture protocols, including our own previously-reported 1 mL solution-based hybridization assay [28]. Although we developed the hybridization method for TB diagnosis and focused our design criteria on the needs of urine cfDNA (ie, short, dilute fragments), we anticipate that our method will be versatile across urine cfDNA applications and may also be useful for other sample types. With the aim of making our method readily accessible to new users and applicable to broader potential applications of hybridization capture, we give a detailed, optimized protocol and outline guidelines for straightforward design of capture probes for new targets.

## Materials and methods

A full step-by-step hybridization capture protocol is posted on protocols.io (http://dx.doi.org/10.17504/protocols.io.bep4jdqw). The protocol below was used for analytical characterization of the hybridization capture method, with experimental details and any modifications for individual experiments noted in the following subsections.

1. **Collect, store, and prepare urine cfDNA**
  - Before urine collection, prepare 15 mL DNA LoBind tubes (Eppendorf, Hamburg, Germany) with 500 μL 0.5 M EDTA and 100 μL 1M Tris-HCl pH 8.
  - Collect urine sample in a sterile container.
  - Immediately after urine collection, add 10 mL of urine to each prepared 15 mL tube and mix by inversion. The final concentration will be 25 mM EDTA and 10 mM Tris-HCl.
  - Freeze at -80°C until analysis.
  - Immediately before analysis, thaw urine at 37°C and mix by inversion.
  - Optionally, test with Fisherbrand 10-SG Urine Reagent Strips (Thermo Fisher Scientific, Waltham, MA, USA).
  - Centrifuge urine for 5 minutes at 8000g to pellet cell debris.
  - Transfer cell-free urine supernatant to new 15 mL DNA LoBind tube (Eppendorf).
2. **Immobilize capture probes on magnetic beads**
  - Vortex Dynabeads MyOne Streptavidin C1 (Thermo Fisher) for 30 seconds to ensure that beads are evenly dispersed in solution.
  - Pipette beads into a 1.5 mL DNA LoBind tube (Eppendorf). Prepare 50 μL (0.5 mg) beads per 10 mL urine sample to be analyzed.
  - Wash beads three times with an equal volume of high salt wash buffer (1M NaCl, 10 mM Tris-HCl pH 8, 0.05% Tween-20).
    - Place beads on magnetic rack for 1 minute; remove and discard supernatant.
    - Add an equal volume of high salt wash buffer (1M NaCl, 10 mM Tris-HCl pH 8, 0.05% Tween-20), vortex for 5 seconds, and briefly spin down.
    - Repeat twice for a total of three washes.
  - Resuspend beads in an equal volume of high salt wash buffer (1M NaCl, 10 mM Tris-HCl pH 8, 0.05% Tween-20).
  - Pre-mix dual biotinylated capture probes BP1 and BP2 (Table 2) in TLE to a final concentration of 50 μM BP1 and 50 μM BP2.
  - Add pre-mixed dual biotinylated capture probes BP1 and BP2 to beads and immediately vortex for 5 seconds. Use 25 pmol of each probe per 50 μL beads (0.5 μL of pre-mixed stock with 50 μM of each probe). If using only a single probe, use 50 pmol per 50 μL beads.
  - Rotate for 15 minutes at room temperature to immobilize probes on beads.
  - Briefly spin down.
  - Wash beads three times with an equal volume of high salt wash buffer (1M NaCl, 10 mM Tris-HCl pH 8, 0.05% Tween-20).
    - Place beads on magnetic rack for 1 minute; remove and discard supernatant.
    - Add an equal volume of high salt wash buffer (1M NaCl, 10 mM Tris-HCl pH 8, 0.05% Tween-20), vortex for 5 seconds, and briefly spin down.
    - Repeat twice for a total of three washes.
  - Resuspend beads in an equal volume high salt wash buffer.
3. **Capture target-specific urine cfDNA by hybridization**
  - Add 2.5 mL 5 M NaCl (final concentration 1 M), 127 μL 10% Tween-20 (final concentration 0.1%) and 50 μL prepared beads to each 10 mL urine sample. If spiking in positive control (Table 2), add it now.
  - Mix well by inversion.
  - Denature for 15 minutes in dry bath with 15 mL tube block preheated to 120°C (urine temperature should reach >90°C).
  - Rotate for 30 minutes at room temperature to hybridize target cfDNA to capture probes.
4. **Wash to remove urine inhibitors and non-target DNA**
  - Centrifuge for 5 minutes at 5000g to pellet beads.
  - Remove and discard all but ~1 mL supernatant using 10 mL serological pipette.
  - Resuspend beads in remaining supernatant and transfer to 1.5 mL DNA LoBind tube (Eppendorf).
  - Place on magnetic rack for 1 minute. Remove and discard supernatant, then remove tube from magnetic rack.
  - Add 1 mL high salt wash buffer (1 M NaCl, 10 mM Tris-HCl pH 8, 0.05% Tween-20) and wash by inverting 10–20 times, or until no bead aggregate is left on tube wall. Do not vortex. Spin down briefly.
  - Place on magnetic rack for 1 minute. Remove and discard supernatant, then remove tube from magnetic rack.
  - Add 1 mL high salt wash buffer (1 M NaCl, 10 mM Tris-HCl pH 8, 0.05% Tween-20) and wash by inverting 10–20 times, or until no bead aggregate is left on tube wall. Do not vortex. Spin down briefly.
  - Place on magnetic rack for 1 minute. Remove and discard supernatant, then remove tube from magnetic rack.
  - Add 1 mL low salt wash buffer (15 mM NaCl, 10 mM Tris-HCl pH 8, no Tween-20) and wash by inverting 10–20 times, or until no bead aggregate is left on tube wall. Do not vortex. Spin down briefly.
  - Place on magnetic rack for 1 minute. Remove and discard supernatant.
  - Spin down again, place on magnetic rack, and remove as much liquid as possible using P20 pipette.
5. **Elute purified target cfDNA**
  - Add 20 μL freshly-prepared 20 mM NaOH, vortex for 5 seconds, and spin down briefly.
  - Place on magnetic rack. Transfer as much supernatant as possible (usually 20–21 μL) directly to qPCR well or to new DNA LoBind tube (Eppendorf). This contains purified target cfDNA. Avoid transferring any beads to qPCR.
  - Partially neutralize with 3.5 μL 100 mM HCl. The qPCR buffer will adjust to final pH, and tolerates slightly basic pH better than acidic pH.
  - Proceed directly to qPCR.
6. **Quantify by qPCR**
  - Analyze the entire hybridization output (approximately 24 μL) from each 10 mL urine sample in a single qPCR well. Each 50 μL reaction should contain 1.25 U OneTaq Hot Start DNA Polymerase (New England Biolabs [NEB], Ipswitch, MA, USA), 1X NEB OneTaq GC Reaction Buffer (NEB; 80 mM Tris-SO_4_, 20 mM (NH_4_)_2_SO_4_, 2 mM MgSO_4_, 5% glycerol, 5% DMSO, 0.06% IGEPAL CA-630, 0.05% Tween-20, pH 9.2), 0.8 mM dNTPs (NEB), 0.4X EvaGreen (Biotium, Fremont, CA, USA), 200 nM forward primer (Table 2), and 200 nM reverse primer (Table 2).
  - Amplify in CFX96 Touch Real-Time PCR Detection System (Bio-Rad Laboratories, Hercules, CA, USA) using an initial denaturation of 94°C for 3 min followed by 45 amplification cycles (94°C for 30s, 64°C for 30s, and 68°C for 1 min).
  - Conduct post-amplification melt analysis from 65°C to 95°C in 0.5°C increments every 5 seconds.
  - Determine C_q_ values at a threshold of 500 RFU, calculate recovered copies using a standard curve (0, 10, 10^2^, 10^3^, 10^4^, and 10^5^ copies positive control) run for each experiment, and verify that melting temperature (T_m_) matches that of expected amplicon.

**Table 2:** Probe, primer, and target sequences.

Oligo	Sequence
Positive control (50 nt)	5’-CGAACCCTGCCCAGGTCGACA**CCATTCAACA**CATAGGTGAGGTCTGCTAC-3’
Reverse complement of positive control (50 nt)	5’-GTAGCAGACCTCACCTATG**TGTTGAATGG**TGTCGACCTGGGCAGGGTTCG-3’
Biotinylated probe #1 (BP1, targets positive control)	5’-/52-Bio/AAAAAAAAAAAAAAAAAAAACAGACCTCACCTATGTGT/3SpC3/-3’
Biotinylated probe #2 (BP2, targets reverse complement)	5’-/52-Bio/AAAAAAAAAAAAAAAAAAAACCCTGCCCAGGTCGA/3SpC3/-3’
Forward primer	5’-CGAACCCTGCCCAGGTCGA-3’
Reverse primer^a^	5’- GTA+GCAGA+CCTCACCTATGTGT-3’
**Length dependence experiment**	
150 nt target	5’-CGAACCCTGCCCAGGTCGACACCATTCAACACATAGGTGAGGTCTGCTACACACCATTCAATTTCATCACTGCCAATACTCCACTCTCATCTACACAACCCATTAGTACCTTACCTCGCTTCCTATCCCAATTCACTTAATCTTAAACCG-3’
80 nt target	5’-CGAACCCTGCCCAGGTCGACACCATTCAACACATAGGTGAGGTCTGCTACACACCATTCAATTTCATCACTGCCAATACT-3’
25 nt target	5’-CCGGCTGTGGGTAGCAGACCTCACC-3’
Biotinylated probe for 25 bp target	5’-/52-Bio/AAAAAAAAAAAAAAAAAAAAGGTGAGGTCTGCTAC/3SpC3/-3’
Primers for ultrashort qPCR of 25 bp target	See Oreskovic *et al*. (2019) [28]
**Comparison of single and dual biotinylated probes**	
Single biotin analog of BP1	5’-/5Biosg/AAAAAAAAAAAAAAAAAAAACAGACCTCACCTATGTGT/3SpC3/-3’

/52-Bio/indicates dual biotin modification; /5Biosg/indicates single biotin modification; /3SpC3/indicates carbon spacer; “+X” indicates LNA base. Target-specific probe binding sequences are underlined. A synthetic spacer region introduced to differentiate the positive control from the endogenous Mycobacterium tuberculosis complex-specific target sequence (IS6110) is bolded (see Table B in S1 Text). All DNA sequences were ordered from Integrated DNA Technologies (IDT; Coralville, IA, USA) with HPLC purification.

^a^ For some early experiments, an older version of the reverse primer without LNA bases was used with a PCR annealing temperature of 58°C. We later added two LNA modifications and increased the annealing temperature to 64°C to improve specificity (see Table A in S1 Text).

### Details of analytical characterization experiments

Experiments were carried out in urine pooled from healthy volunteers (approved by the University of Washington Human Subjects Division, IRB #48840). Participants gave written consent. For all experiments, the total capture probe concentration was kept constant at 50 pmol probe per 50 μL beads. For experiments targeting dsDNA (positive control and reverse complement), both probes BP1 and BP2 were used (25 pmol BP1 and 25 pmol BP2 per 50 μL beads) unless otherwise specified. For experiments targeting single-stranded DNA (ssDNA) (positive control only), only probe BP1 was used (50 pmol per 50 μL beads) unless otherwise specified. Some experiments used an older version of the reverse primer without LNA modifications and a lower qPCR annealing temperature of 58°C. Using these conditions, we occasionally observed late, probe-independent nonspecific amplification of residual human genomic DNA (see S1 Text). We updated the reverse primer design to include LNA substitutions to increase its melting temperature (T_m_) to match that of the forward primer and increased the qPCR annealing temperature to 64°C (see Table A in S1 Text). We found that this change minimized nonspecific amplification while maintaining high qPCR efficiency. Unless otherwise noted, we used the LNA-modified reverse primer and an annealing temperature of 64°C. For all experiments, we maintained laboratory practices intended to limit contamination, including separating pre- and post-PCR rooms, regularly decontaminating work surfaces and pipettes, using sterile filtered pipette tips, and aliquoting reagents into single-use volumes.

#### Percent recovery, representative calibration curves, and limit of detection

To determine the purification efficiency, 10^3^ copies of 50 bp dsDNA (Table 2) were spiked into 10 mL urine, extracted by hybridization, and amplified by qPCR (n = 6 independent experiments processed on separate days). Purification efficiency was calculated as the percent of DNA spiked into urine that was recovered and detected by qPCR. Representative calibration curves for DNA recovered by hybridization were generated by spiking 0, 5, 10, 10^2^, 10^3^, 10^4^, or 10^5^ copies (0, 0.5, 1, 10, 10^2^, 10^3^, or 10^4^ copies/mL) of 50 bp dsDNA into 10 mL urine, extracting by hybridization, and amplifying by qPCR (n = 3). Representative calibration curves for the qPCR standards were generated by adding 0, 5, 10, 10^2^, 10^3^, 10^4^, or 10^5^ copies of 50 bp dsDNA directly into qPCR. The limit of detection was verified by spiking 0 or 5 copies (0.5 copies/mL) of 50 bp dsDNA into 10 mL urine, extracting by hybridization, and amplifying by qPCR (n = 6 technical replicates from the same experiment).

#### Length dependence

To measure the recovery across fragments of different lengths, 10^3^ copies of 25, 50, 80, or 150 nt ssDNA (Table 2) were spiked into 10 mL urine and extracted by hybridization using BP1 (50, 80, 150 nt targets) or the 25 nt capture probe (25 nt target) listed in Table 2 (n = 3 independent experiments with 3 technical replicates per experiment). The 80 nt and 150 nt fragments were amplified using the same primers and qPCR conditions as the 50 nt fragment. For this experiment, the reverse primer without LNA modifications was used. The 25 nt fragment was amplified using ultrashort qPCR as described previously [28].

#### Tolerance to varied urine conditions

To test the effects of variations expected in clinical urine samples, 10^3^ copies of 50 nt ssDNA were spiked into 10 mL PBS with varied pH (5, 6, 7, 8), non-target DNA (0, 1, 10 μg sheared salmon sperm DNA), and salt (13.7, 137, 500 mM NaCl) conditions, extracted by hybridization, and amplified by qPCR using the reverse primer without LNA modifications (n = 1).

#### Resistance to qPCR inhibition

To test for qPCR inhibition, eluate extracted from 10 mL urine (no added target) was spiked into qPCR containing 10^3^ copies of 50 nt ssDNA (0, 1, 5, 10, or 20 μL eluate in 50 μL qPCR, for final 0%, 2%, 10%, 20%, or 40% eluate, respectively) (n = 3 technical replicates from the same experiment). Amplification was carried out using the reverse primer without LNA modifications. An increase in quantification cycle (C_q_) was used to indicate qPCR inhibition.

#### Comparison of one and two probe systems

To compare recovery of dsDNA using the one and two probe systems, 1000 copies of 50 bp dsDNA were spiked into 10 mL urine, extracted by hybridization, and amplified by qPCR. For the single probe system, 50 pmol of capture probe (BP1 or BP2) was used per 50 μL beads (n = 3 technical replicates from the same experiment). For the two probe system, 25 pmol of each probe (BP1 and BP2) was used per 50 μL beads (n = 3 technical replicates from the same experiment). To confirm that introduction of a second probe had no effect on the recovery of the opposite probe, 1000 copies of 50 nt ssDNA (positive control or reverse complement) were spiked into 10 mL urine, extracted by hybridization (50 pmol BP1 or BP2 or 25 pmol each of BP1 and BP2), and amplified by qPCR (n = 3 technical replicates from the same experiment). To determine if re-hybridization of complementary target strands affected recovery, 1000 copies of 50 nt ssDNA or 50 bp dsDNA were spiked into 10 mL urine, extracted by hybridization (50 pmol BP1 or 50 pmol BP2), and amplified by qPCR (n = 3 technical replicates from the same experiment). For these experiments, the reverse primer without LNA modifications was used.

#### Comparison of single and dual biotinylated capture probes

To determine the dependence on probe concentration during immobilization, beads were functionalized with 5, 25, 50, 125, 250, or 500 pmol dual biotinylated BP1 or the analogous single biotinylated probe (Table 2) per 50 μL beads. 10^3^ copies of 50 nt ssDNA were spiked into 10 mL urine, extracted by hybridization, and amplified by qPCR (n = 3 independent experiments processed on separate days). To test thermostability, beads were functionalized with 50 pmol dual biotinylated BP1 or the analogous single biotinylated probe per 50 μL beads and used to extract 10^3^ copies of 50 nt ssDNA spiked into 10 mL urine. Beads were either added to the sample prior to denaturation at >90°C for 15 minutes, or after the sample was denatured and cooled to room temperature (n = 3 independent experiments processed on separate days). To test tolerance to free biotin, beads were functionalized with 50 pmol dual biotinylated BP1 or the analogous single biotinylated probe per 50 μL beads. Before hybridization, functionalized beads were incubated for 15 minutes at room temperature with or without 1 μM free D-biotin and washed three times with high salt wash buffer (1 M NaCl, 10 mM Tris-HCl pH 8, 0.05% Tween-20). Beads were then used to extract 10^3^ copies of 50 nt ssDNA spiked into 10 mL urine (n = 3 independent experiments processed on separate days). For these experiments, the reverse primer without LNA modifications was used.

#### Demonstration of multiplexing feasibility

To demonstrate the feasibility of multiplexing with more than two probes, the ratio of target-specific probes BP1 and BP2 (targeting the *M*. *tuberculosis* insertion element IS6110) to non-target probes (targeting the tuberculosis direct repeat (DR) region: 5’-/52-Bio/AAAAAAAAAAAAAAAAAAAATCAGACCCAAAACCCC/3SPC3-3’ and 5’-/52-Bio/AAAAAAAAAAAAAAAAAAAATCCGTCCCCTCTCGG/3SPC3-3’) was varied while keeping the total probe concentration constant at 50 pmol per 50 μL beads. For each condition, 10^3^ copies of 50 bp dsDNA were spiked into 10 mL urine, extracted by hybridization, and amplified by qPCR (n = 3 technical replicates from the same experiment). Conditions tested represented one double-stranded target (100:0 ratio of IS6110 probes to DR probes), two double-stranded targets (50:50), five double-stranded targets (20:80), and ten double-stranded targets (10:90). DR template DNA was also spiked in each condition to account for any effects due to binding of other targets (0, 1000, 4000, or 9000 copies, respectively).

### Statistical methods

When appropriate (*i*.*e*., when replicates were carried out across multiple, independent experiments), conditions were compared using one-way ANOVA with Tukey’s honestly significant difference post hoc test. Statistical analysis was conducted using GraphPad Prism v8.1.2 (San Diego, CA, USA) with a significance level of 0.05.

## Results and discussion

### Overview of hybridization capture method and probe design

**Fig 2:**
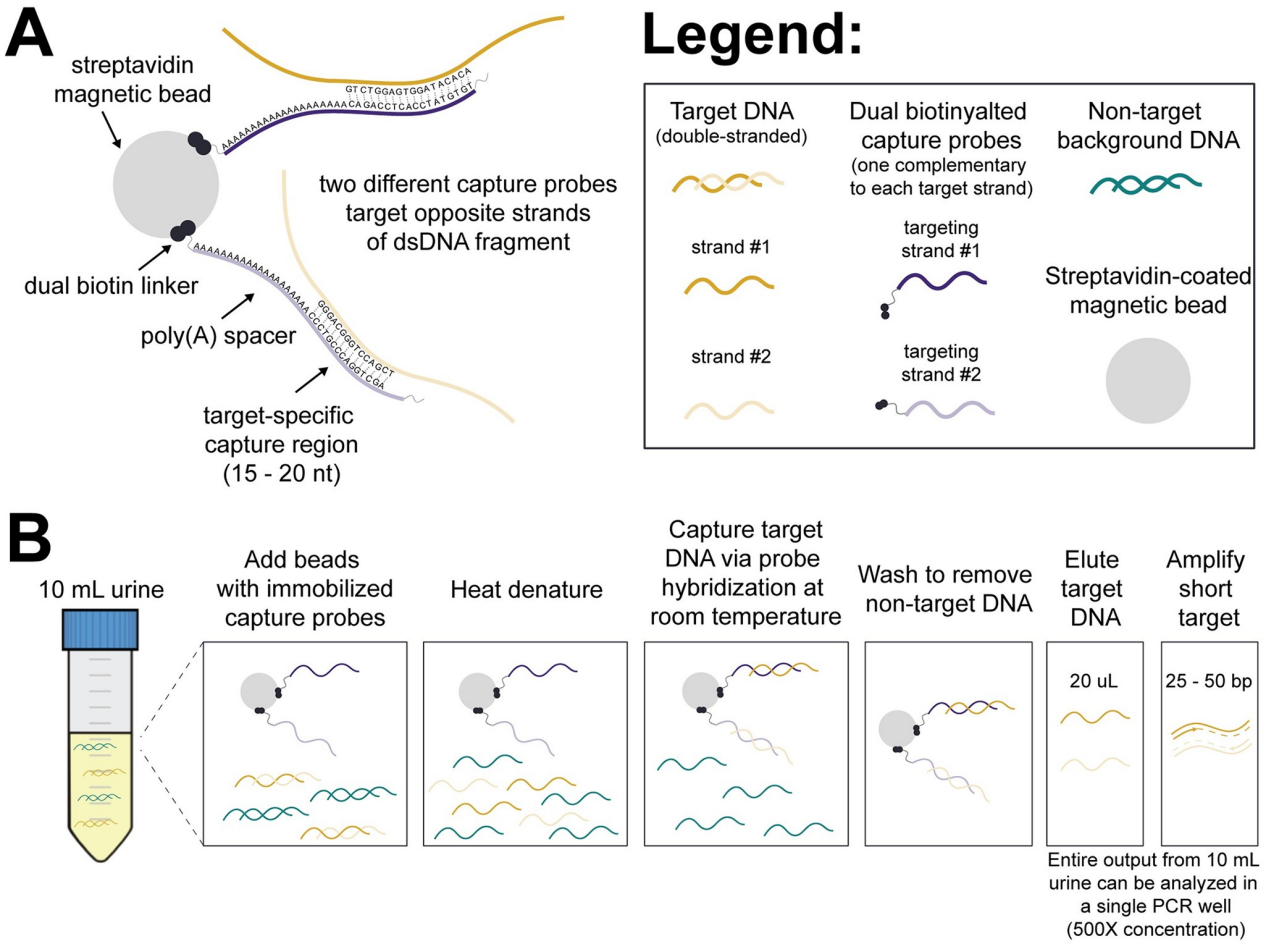
Hybridization capture probe design and procedure. **(A)** Schematic illustrating hybridization capture probe design. Two short, sequence-specific probes, one complementary to each strand of the double-stranded target region, are immobilized on streptavidin-coated magnetic beads via dual biotin linkers. **(B)** Overview of hybridization capture procedure. Beads with pre-immobilized capture probes are added to 10 mL urine (along with 1 M NaCl and 0.1% Tween-20), heat denatured (15 minutes at >90°C), hybridized to target DNA (30 minutes at room temperature), and washed to remove non-target DNA (2X high-salt wash, 1X low-salt wash). Purified target-specific DNA is eluted under basic conditions, neutralized, and amplified by short-target qPCR.

The hybridization capture probe design is shown in Fig 2A. Two unique capture probes are used to hybridize to both strands of the dsDNA target region. Each probe contains a short sequence-specific binding region (15–20 nt) complementary to the target of interest and a 5’ dual biotin modification for immobilization on streptavidin magnetic beads. The probes also contain a 20 nt poly(A) spacer between the dual biotin modification and target-specific binding sequence to reduce steric hindrance during hybridization and a 3’ C3 blocker to prevent extension of any residual probes during qPCR. As discussed in later sections, both the dual biotin modification of probes and the two-probe system for recovery of both strands of dsDNA are critical design elements for the strong analytical performance of our method.

An overview of the hybridization capture procedure is given in Fig 2B. Dual biotinylated capture probes complementary to the target of interest are immobilized on streptavidin-coated magnetic beads prior to hybridization. The functionalized beads are added to a 10 mL urine sample, along with NaCl (1M) and Tween-20 (0.1%). A relatively high effective probe concentration (2 nM of each probe) helps drive rapid hybridization. Urine is denatured at >90°C for 15 minutes before the DNA target sequence is captured via hybridization to probes at room temperature for 30 minutes. The beads are concentrated by centrifugation, then washed on a magnetic rack, twice with a high salt buffer and once with a low salt stringency buffer, to remove urine and non-target DNA. The purified target-specific DNA is eluted under basic conditions, neutralized, and detected by short-target qPCR. The entire purified output (20 μL) from 10 mL urine can be analyzed in a single qPCR well, resulting in 500X concentration from the original sample volume and minimizing dilution to enable detection of low concentration targets.

### Analytical performance of hybridization capture method

**Fig 3:**
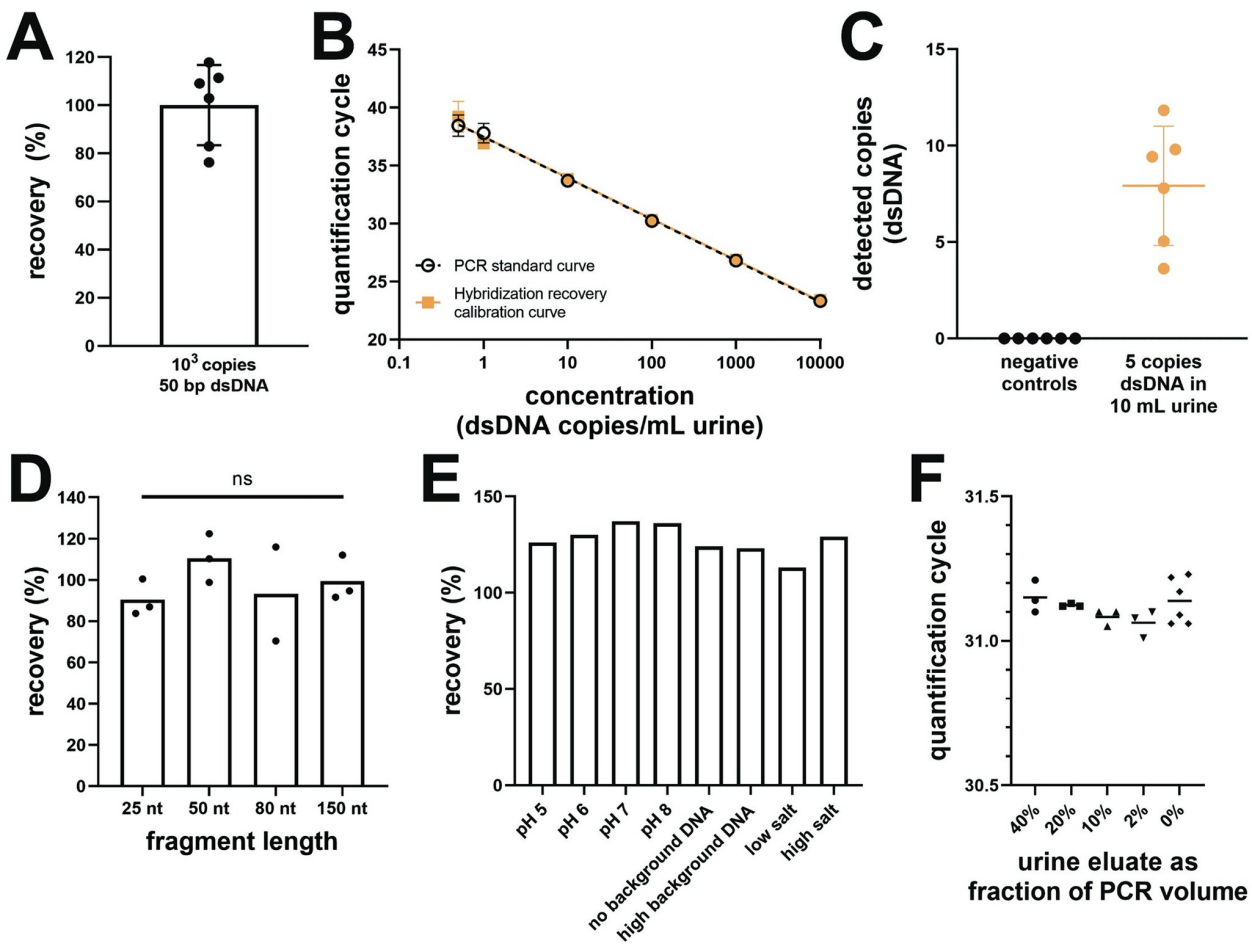
Analytical performance of the hybridization capture method. **(A)** The hybridization capture method recovers nearly all target-specific dsDNA (10^3^ copies of 50 bp positive control) spiked into 10 mL urine (mean ± SD, n = 6 independent experiments processed on different days). **(B)** Representative calibration curves for DNA recovered by hybridization and qPCR standards overlap across concentrations from 0.5–10^4^ copies/mL 50 bp dsDNA in urine (mean ± SD, n = 3). **(C)** The hybridization method reliably distinguishes 5 copies of 50 bp dsDNA spiked into 10 mL urine (0.5 copies/mL) from negative controls (mean ± SD, n = 6). **(D)** Hybridization has high recovery across fragment lengths from 25–150 nt (mean, n = 3 independent experiments processed on different days, 10^3^ copies ssDNA input). Each dot represents the mean of 3 technical replicates performed for each independent experiment. One experiment was excluded for the 80 nt fragment length due to a calculated recovery of >200% caused by delay in amplification of the PCR standards. ns indicates not significant (one-way ANOVA with post-hoc Tukey test). **(E)** Hybridization is tolerant to variations expected in clinical urine samples, including pH (5–8), non-target DNA (0–10 μg), and salt (0–500 mM) (n = 1, 10^3^ copies 50 nt ssDNA input). **(F)** Hybridization enables amplification of entire output from 10 mL urine in a single qPCR well without inhibition. Eluate extracted from pooled urine (no added target) was spiked into qPCR containing a constant target concentration (10^3^ copies 50 nt ssDNA). Resistance to qPCR inhibition is indicated by the lack of increase in quantification cycle (C_q_) despite increasing fraction of eluate (mean, n = 3).

Our hybridization capture method recovers 100.1% (95% CI: 82.6–117.6%) of DNA spiked into 10 mL urine (Fig 3A). The calculated percent recovery is occasionally >100% due to variability in qPCR quantification when using a unique standard curve for each experiment (see S1 Fig), but centers near 100% across multiple independent experiments. Fig 3B shows that representative calibration curves for DNA spiked into 10 mL urine and recovered by hybridization and DNA spiked directly into qPCR are visually indistinguishable, illustrating near complete recovery of DNA from urine across a range of concentrations. Using our hybridization capture method, we can reliably distinguish 5 copies of dsDNA spiked into 10 mL urine from negative controls (Fig 3C). Our protocol improves the limit of detection (≤0.5 copies/mL dsDNA, or 1 copy/mL ssDNA, approaching the detection limit of qPCR) compared to previous hybridization capture protocols, which had, at best, analytical sensitivities in the range of 5–100 copies/mL (Table 1). Our protocol also increases the input sample volume to 10 mL from a previous maximum of 1 mL (Table 1). Testing in our lab indicates that the increase in sample volume was crucial for detection of TB-specific cfDNA in urine from patients with pulmonary TB. We have completed a study to determine the diagnostic accuracy of our hybridization assay in clinical urine samples from patients with pulmonary TB, the results of which will be published separately to build upon the analytical characterization reported here (manuscript in preparation).

Critically for urine cfDNA, which is made up primarily of short fragments (majority <100 bp) [8,15–17], our hybridization capture method is not dependent on DNA fragment length and maintains high recovery across fragments from 25–150 bp (Fig 3D). Combined with an ultrashort hairpin qPCR design [28], we can capture and amplify fragments as short as 25 bp with high efficiency (84% recovery). In contrast, conventional DNA extraction based on adsorption of DNA to silica under chaotropic conditions is dependent on DNA fragment length, with reduced recovery of shorter fragments. DNA adsorption to silica is driven by hydrophobic interactions between dehydrated silica and DNA surfaces and hydrogen bonding between silica and the DNA backbone [25], both of which are dependent on DNA fragment length. We previously reported that several silica-based methods for urine cfDNA extraction, both published lab-based protocols and commercially-available kits, have reduced recovery of short DNA fragments [28]. We determined that the frequently-used Wizard silica resin method, which was used in the first study demonstrating the presence of cfDNA in urine [1], had low recovery (<35%) of 40–150 bp fragments, with a further drop in recovery for 25 bp fragments (<2%) [28]. Even the best-performing method that we identified (Q Sepharose anion exchange resin [9]), which had moderate recovery (63–75%) of 40–150 bp fragments, had low recovery of the shortest 25 bp fragment (9%) [28].

Hybridization capture is expected to perform well across varied clinical urine samples due to the insensitivity of DNA hybridization to properties that vary in urine samples. Specifically, we have demonstrated tolerance to a wide range of pH (5–8), non-target DNA (0–10 μg), and salt (0–500 mM) conditions (Fig 3E). Particularly notable is the robust performance for low concentrations of non-target background DNA, as total urine cfDNA concentrations are expected to be low (<1–200 ng/mL) [1,10,23]. In contrast, silica-based DNA extraction may perform poorly for low DNA concentrations, which is why many silica-based kits require the addition of carrier nucleic acids to maintain high yields for low-concentration samples. In the presence of high chaotrope concentrations, a fraction of DNA may bind irreversibly to the silica surface due to strong hydrophobic interactions [26,27]. For high-concentration samples, this loss may be trivial, but for low-concentration samples like urine cfDNA the unrecoverable DNA may make up a significant fraction of the input [26,27]. In our previous comparison of urine cfDNA extraction methods, we found that the Wizard silica resin method was highly dependent on sample composition, and was thus likely to fail in a subset of clinical urine samples with high pH and/or low non-target DNA concentrations [28].

Our hybridization capture protocol also efficiently removes qPCR inhibitors in urine that may affect downstream amplification, enabling amplification of cfDNA from 10 mL urine in a single qPCR well without inhibition (Fig 3F). Although not a necessity for dilute urine cfDNA, sequence-specific hybridization capture is also beneficial for samples where high concentrations of co-extracted non-target DNA can act as an inhibitor of downstream amplification (eg, stool, sputum, whole blood) [44,56,58]. Non-target DNA amounts of 2–10 μg or higher have been reported to inhibit amplification [44,56,60], but can be effectively removed by hybridization (Fig 3E). While we have not tested sample types besides urine here, we anticipate that our hybridization method could also be broadly useful for samples with a high background of non-target DNA.

### Two probe system enables recovery of both strands of double-stranded DNA

**Fig 4:**
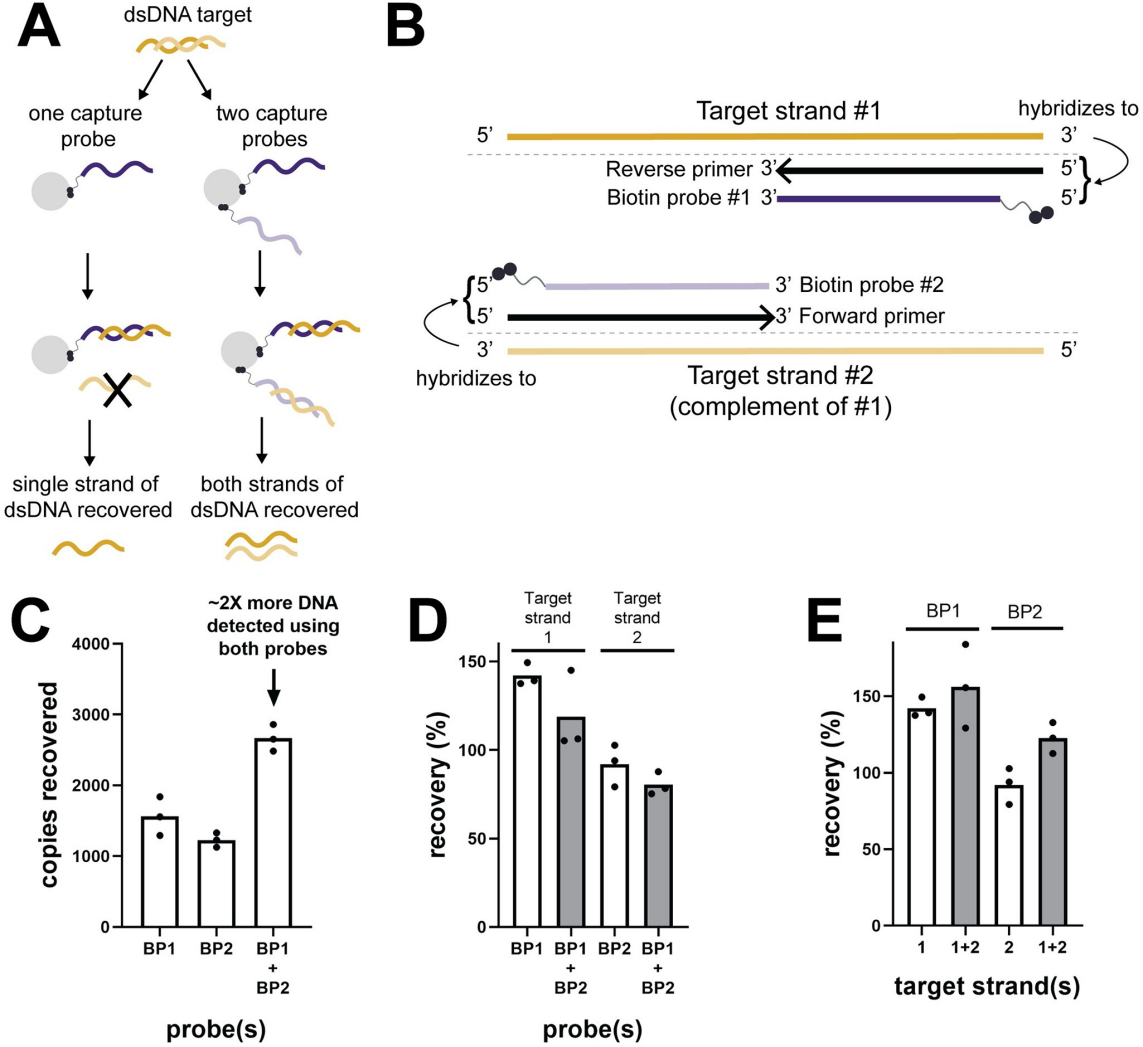
Two probe system enables recovery of both strands of double-stranded DNA. **(A)** Rationale for two-probe system. When using a single capture probe (left column), only one strand of the dsDNA target is recovered by hybridization; the other strand is discarded. When a second capture probe is added to target the complementary strand (right column), both strands of dsDNA are recovered, doubling the concentration of detectable DNA. **(B)** Schematic of two-probe system design. To minimize target footprint, probes are truncated versions of the forward and reverse qPCR primers (15–20 nt). Probes hybridize to different sub-regions of the target sequence so that opposite probes do not hybridize to each other. **(C)** Using both capture probes results in an approximate 2-fold increase in detected dsDNA compared to either single probe (mean, n = 3 technical replicates from the same experiment; 10^3^ copies 50 bp dsDNA input). Each target strand was quantified by a different standard curve, so the minor difference observed in calculated percent recovery for BP1 and BP2 is unlikely to be meaningful (see S1 Fig; same applies for panels D-E). **(D)** Addition of a second probe does not affect recovery of ssDNA by the opposite probe (mean, n = 3 technical replicates from the same experiment; 10^3^ copies 50 nt ssDNA input). **(E)** Re-hybridization of complementary dsDNA target strands is negligible compared to probe-target hybridization and does not affect recovery (mean, n = 3 technical replicates from the same experiment; 10^3^ copies 50 nt ssDNA or 50 bp dsDNA input). Because plots in this figure show technical replicates from the same experiment, the data are not independent and statistical analysis was not conducted.

Because hybridization targets single-stranded DNA, it typically only captures half of available target molecules (ie, DNA strands) compared to non-specific extraction methods such as silica adsorption that capture both strands of double-stranded DNA. To overcome this limitation, we designed two capture probes, one complementary to each strand of the dsDNA target (Fig 4A and 4B). As shown in Fig 4C, the two-probe system increases detected copies by approximately two-fold compared to either single probe alone by enabling recovery of both strands of dsDNA. This design element is especially important for low-concentration targets where the number of purified target copies may be near the limit of detection of qPCR. By recovering both strands of the dsDNA target, we improve sensitivity by doubling the number of potential amplicons for qPCR and increasing the likelihood of successful detection in low-concentration clinical samples. The addition of a second capture probe does not affect recovery of the first probe when the total probe concentration is held constant (Fig 4D). Re-hybridization of dsDNA target stands is thermodynamically favored over hybridization to capture probes, but the high concentration of capture probes kinetically favors probe-target hybridization. Re-hybridization of dsDNA is negligible and does not affect target recovery after 30 minutes of hybridization at room temperature (Fig 4E).

### Advantages of dual biotinylated capture probes

**Fig 5:**
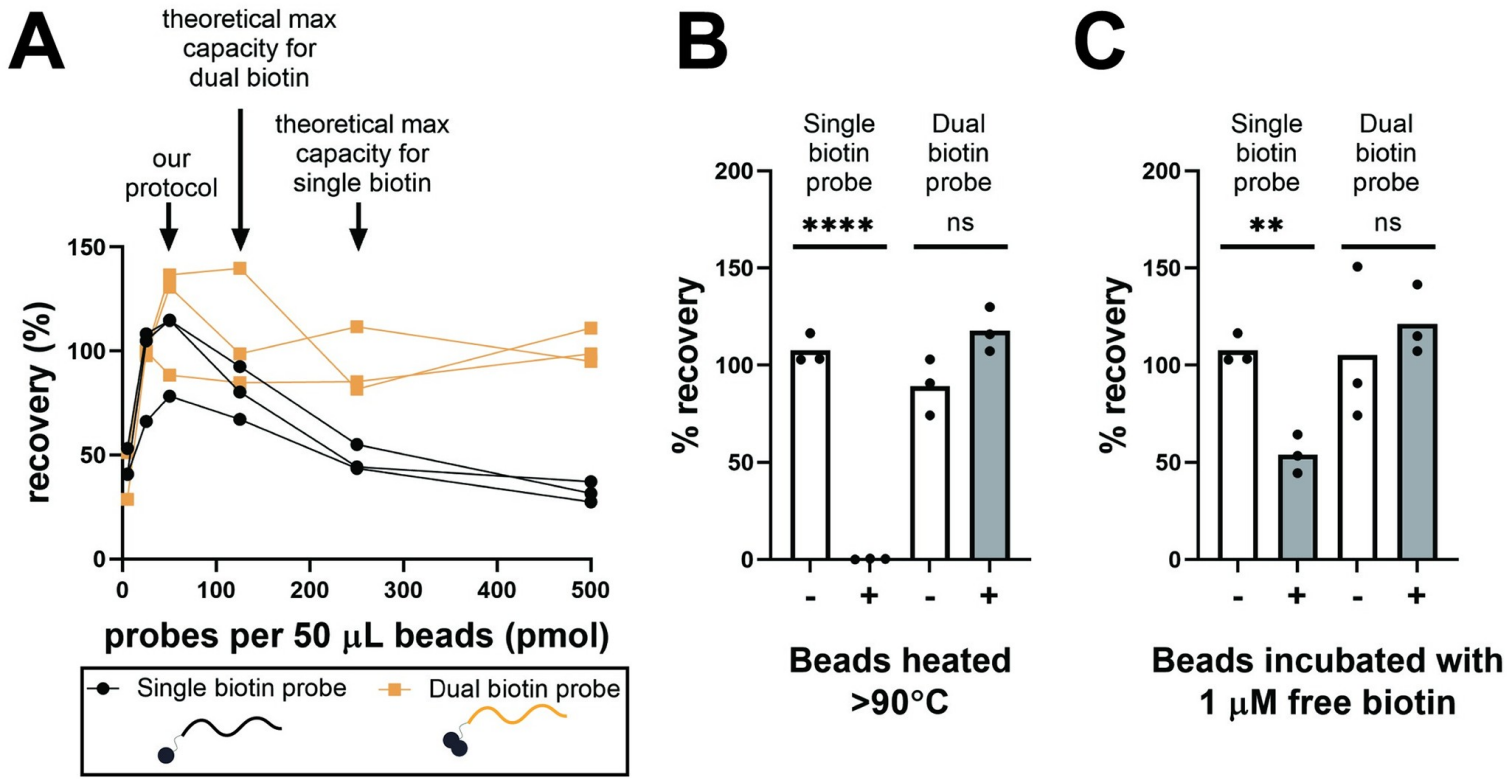
Dual biotinylated capture probes improve hybridization performance compared to single biotinylated capture probes. **(A)** Dual biotinylated probes (in orange) facilitate optimal spacing and density of probes on the bead surface, increasing recovery and reducing reliance on the probe concentration used during bead functionalization compared to single biotinylated probes (in black) (n = 3 independent experiments on different days; 10^3^ copies 50 nt ssDNA input). **(B)** Beads functionalized with dual biotinylated probes are thermostable up to at least 90°C, while beads functionalized with single biotinylated probes lose function after heating to 90°C. Grey columns labeled as “+” were heated to >90°C for 15 minutes prior to hybridization; white columns labeled as “-”were unheated controls (mean, n = 3 independent experiments on different days; 10^3^ copies 50 nt ssDNA input). **(C)** Beads functionalized with dual biotinylated probes tolerate incubation with free biotin, while beads functionalized with single biotinylated probes have reduced recovery after incubation with free biotin. Grey columns labeled as “+” were incubated for 15 minutes with 1 μM free biotin; white columns labeled as “-”were controls not incubated with free biotin (mean, n = 3 independent experiments on different days; 10^3^ copies 50 nt ssDNA input). ** indicates P value of 0.001 to 0.01, **** indicates P value of <0.0001, ns indicates not significant (one-way ANOVA with post-hoc Tukey test).

Dual biotinylated probes, which have two biotin molecules in series at the 5’ end of the probes, are a key design element of our hybridization protocol that help ensure its high sensitivity and robust performance. As shown in Fig 5A, dual biotinylated probes increase recovery and reduce reliance on the probe concentration used during probe immobilization compared to single biotinylated probes. When we varied the amount of probes added to beads during the probe immobilization step, single biotinylated probes had only a narrow peak in probe concentration (25–50 pmol probe per 50 μL beads) that led to maximum recovery. Higher probe concentrations resulted in reduced recovery, with 50% or less recovery for probe concentrations at or above that expected to saturate beads (estimated as 250 pmol per 50 μL beads for single biotinylated probes). Dual biotinylated probes, on the other hand, had high recovery across a broad range of probe concentrations (25–500 pmol probe per 50 μL beads). Even when dual biotinylated probes were added at concentrations beyond that expected to saturate beads (estimated as 125 pmol per 50 μL beads for dual biotinylated probes), hybridization recovery remained near 100%.

Probe density has a significant, well-documented impact on the extent of DNA hybridization on surfaces, due to both thermodynamic and kinetic effects [61,62]. If the probe density is too low, the kinetics of hybridization will be slow, resulting in low recovery, as is likely the case in Fig 5A for both single and dual biotinylated probes at 5 pmol per 50 μL beads. If the probe density is too high, steric hindrance and electrostatic repulsion near the bead surface can reduce both the rate of hybridization and the final hybridization efficiency [61,62], as is likely the case in Fig 5A for single biotinylated probes at ≥125 pmol probe per 50 μL beads. Several studies have observed a similar density-dependent peak in hybridization efficiency for immobilized probes [61,63–65], including on magnetic beads [66], with hybridization efficiency reported to drop at probe densities greater than 4–5 x 10^12^ probes/cm^2^ [61,63]. We selected 50 pmol dual biotinylated probe per 50 μL beads as the concentration to use during coupling, which corresponds to a probe density of 1.9–2.7 x 10^12^ probes/cm^2^ (370–526 Å^2^/probe) on the bead surface, which is within the optimal range for rapid, efficient hybridization. Importantly, heterogeneity of probe distribution across the surface can also lead to sub-optimal recovery [67]. Dual biotin modification of capture probes likely helps avoid steric hindrance both by limiting overall maximum probe density (by saturating streptavidin at a lower probe density) and facilitating optimal spatial distribution of probes on the bead surface (by limiting to only one or two probes per tetrameric streptavidin molecule). The practical implication of this phenomenon is that non-optimal bead:probe ratios during probe immobilization are unlikely to compromise assay performance if dual biotinylated probes are used, eliminating the need for careful tuning of probe density and reducing variability between bead batches. We found only a single instance of dual biotinylated probes used previously for hybridization-based sample preparation, which, although overall recoveries were lower (<30%), reported a similar effect for reduced dependence on probe concentration relative to single biotinylated probes [53]. The authors measured dual biotinylated probe density at saturation and confirmed that it was half of that of single biotinylated probe density at saturation, as expected [53].

In addition to moderating probe density, the dual biotin modification is advantageous because it increases thermostability of the bead-probe linkage. Beads functionalized with dual biotinylated probes maintained recovery after heating at >90°C for 15 minutes, while beads functionalized with single biotinylated probes lost all function after heating (Fig 5B). Dual biotinylated probes likely increase biotin-streptavidin thermostability by more fully saturating streptavidin with biotin while still maintaining an optimal probe density. Saturation with biotin increases the T_m_ of thermally-induced denaturation of streptavidin from 75°C to 112°C [68]. The increase in thermostability is incremental, with streptavidin 50% saturated with biotin showing two distinct thermogram peaks [68]. Dual biotinylated probes have been used previously to improve biotin-streptavidin thermostability during heat elution [53,69] and enable PCR thermocycling without leeching of biotinylated DNA from streptavidin beads [53,70]. For our purposes, enhanced streptavidin thermostability means that beads can be added to urine before the denaturation step without loss in performance. Denaturation of beads functionalized with single biotinylated probes, or adding beads functionalized with single biotinylated probes to heated samples immediately after denaturation prior to cooling, results in reduced recovery due to loss in function as streptavidin denatures. Cold shock, which is commonly used to cool denatured samples while minimizing DNA renaturation, could avoid this problem for single biotinylated probes but is not effective for large volume samples. Allowing for pre-heating of beads may also improve availability of surface-immobilized probes, leading to an increase in hybridization recovery [61].

Saturating unbound streptavidin with biotin after probe immobilization may achieve a similar improvement in thermostability, but single biotinylated probes are negatively affected by the presence of high biotin concentrations. Although the streptavidin-biotin association is strong, some dissociation does occur [71]. In the presence of high concentrations of free biotin, competitive dissociation may displace bound biotinylated probes over time. We observed reduced recovery after incubating beads functionalized with single biotinylated probes with 1 μM free biotin for 15 minutes, but when using dual biotinylated probes, the difference in recovery with and without biotin incubation disappeared (Fig 5C). Endogenous biotin is a known interferent in streptavidin-based diagnostic assays [72], so the tolerance of dual biotinylated probes to high concentrations of free biotin also eliminates the risk of compromised results in urine samples with high endogenous biotin concentrations. Covalently-immobilized probes may also improve thermostability and tolerance to endogenous biotin, but it is difficult to tailor the probe density and spacing during the conjugation reaction. Using dual biotinylated probes with streptavidin-coated magnetic beads retains the ability to carefully control probe density, one of the key advantages of biotin-streptavidin immobilization over covalent conjugation, while also maintaining thermostability and tolerance to endogenous biotin, characteristics which are lacking for single biotinylated probes.

### Comparing surface-based and solution-based hybridization capture

We have previously reported a solution-based hybridization assay, which had similar performance characteristics (73–84% recovery of 25–150 bp DNA) to the surface-based assay described here, but was limited to a 1 mL sample volume [28]. Because capture probes were hybridized to target cfDNA prior to immobilization on beads, an excess of beads was required for the solution-based method to avoid competition of biotinylated probes with endogenous biotin in urine. The beads make up the majority of the assay cost, so scaling up solution-based hybridization (~$15/mL) for a larger 10 mL urine sample was cost-prohibitive. Switching to the surface-based hybridization approach described here enabled us to avoid competition with endogenous biotin by pre-immobilizing probes on beads prior to hybridization, reduce bead consumption and cost per milliliter of sample analyzed ($0.95/mL), and scale up the reaction volume to 10 mL. With minor modifications, we anticipate that our current protocol could be further scaled up to analyze 20–40 mL samples without a significant increase in cost per sample. While the kinetics of surface-based hybridization are slower than the kinetics of solution-based hybridization, using microparticles rather than planar surfaces helps to mitigate the effect on reaction rate [66]. With our optimized protocol, surface-based hybridization remains rapid, with near complete hybridization in ≤30 minutes at room temperature. Increasing the hybridization temperature or time does not lead to an improvement in performance (S2 Fig).

Based on our experience, we generally recommend surface-based hybridization over solution-based hybridization, particularly when large sample volumes are required. Surface-based hybridization with dual biotinylated capture probes eliminates the risk of biotin interference while remaining cost-effective for large sample volumes, while solution-based hybridization may be compromised by high endogenous biotin concentrations and is more expensive. Assay time and percent recovery using the two approaches are comparable, as long as conditions are optimized. Specifically, optimal probe density is critical for success with surface-based hybridization, as discussed above.

### Designing capture probes for application to new targets

Designing capture probes for new targets is straightforward. To reduce target footprint and minimize design effort, we recommend using truncated versions of the qPCR primers (see Fig 4B). This approach will also automatically limit secondary structure, which should be minimal in well-designed primers. Nucleotides should be removed from the 5’ end of the primers until a length of 15–20 nt is reached and the melting temperatures of the two probes are similar (as a rule of thumb, ~60°C as calculated in hybridization conditions of 1 M NaCl and 2 nM probe). In addition to the target-specific binding region, probes should contain a 5’ dual biotin modification, a 20 nt poly(A) spacer between the biotin modification and the target-specific binding region, and a 3’ carbon spacer, as described in Table 2 and Fig 2A. Capture probes should be ordered with HPLC purification to ensure full-length probes. The hybridization method should have high, robust recovery independent of probe sequence. In our experience, implementation of new capture probes does not require design iterations or assay optimization. For example, by following the above design rules, we designed a capture probe targeting the direct repeat (DR) sequence of *M*. *tuberculosis* that had >90% recovery and ≤1 copy/mL sensitivity.

In general, existing qPCR assays should work in combination with hybridization capture. For urine cfDNA, it is important to keep the qPCR amplicon as short as possible (ideally ≤50 bp) to maximize the fraction of cfDNA fragments that contain both primer sites and can be successfully amplified. Because the exact eluate volume varies slightly across samples, we found that partial neutralization leaving the eluate slightly basic had the least effect on downstream amplification. In optimization experiments where we controlled the eluate pH, the qPCR tolerated slightly basic eluate better than slightly acidic eluate. For new targets, we recommend using qPCR conditions with high buffering capacity (for example, 80 mM Tris-SO_4_ used here). If amplification inhibition is observed, we suggest supplementing the qPCR buffer with additional Tris-HCl. To easily identify contamination and prevent false positives during clinical testing, we also recommend adding a 10–20 bp spacer region between the primer binding sites on your target of interest to design a synthetic positive control that can be distinguished from the native target of interest by post-amplification melt analysis (see Table B in S1 Text).

To summarize, the suggested selection criteria for target, primer, and probe sequences are as follows:

1. The target sequence should be selected for specificity to the target of interest. Targeting multi-copy genomic elements may help improve clinical detection sensitivity.
2. The primers should be designed to amplify as short as possible (40–50 bp maximum for urine cfDNA) of a region within the target sequence while maintaining specificity.
3. The capture probe sequences should be truncated versions of the primer sequences, with bases removed from the 5’ end to reach lengths of 15–20 nt long and melting temperatures of ~60°C at a concentration of 2 nM probe in 1 mM NaCl. A dual biotin modification and 20 nt poly(A) spacer should be added to the 5’ ends, and a C3 spacer should be added to the 3’ ends.

### Demonstration of multiplexing feasibility

To demonstrate the potential multiplexing capability of hybridization capture and estimate the maximum number of probes (*i*.*e*., the minimum concentration per probe) that maintains high recovery, we varied the ratio of target to non-target probes while keeping the total probe concentration constant (S3 Fig). We found that recovery remained the same for conditions representing up to four probes (two probe sets targeting two dsDNA targets or four individual probes targeting four ssDNA targets). Recovery dropped moderately to 63% of the original for 10 probes (targeting five dsDNA targets or 10 ssDNA targets) and was substantially reduced to 26% of the original for 20 probes (targeting 10 dsDNA targets or 20 dsDNA targets). Small-scale multiplexing is possible using our current protocol, and larger-scale multiplexing may be improved through additional optimization specifically for multiplexed capture, such as a longer hybridization time or increased bead volume.

### Potential applications, limitations, and future work

We developed this hybridization capture protocol with the goal of improving the sensitivity of TB diagnosis from urine cfDNA, but we expect that it will be versatile across urine cfDNA applications such as infectious disease diagnosis [4,5], cancer detection [1–3], organ transplant monitoring [6,7], and fetal genetic screening [1,8]. It may also be broadly useful for targeting cfDNA in other sample types (eg, plasma, cerebral spinal fluid, saliva) or for applications beyond cfDNA. Hybridization is likely to be especially beneficial for removing high concentrations of non-target background DNA, which can inhibit downstream amplification and contribute to non-specific amplification [44,56,58], and for concentrating and detecting dilute targets from large volume samples. Potential applications that may benefit from the sequence-specific nature of hybridization include infectious disease diagnosis from whole blood, sputum, and stool, all of which contain high concentrations of amplification inhibitors, including host DNA, and environmental monitoring, which faces similar challenges of dilute targets in large sample volumes with an excess of non-target DNA. Sequence-specific hybridization capture has been previously demonstrated to increase the analytical sensitivity of PCR-based detection of TB in pleural fluid (by 10- to 100-fold) [44] and in sputum [58,59], and to increase the amount of background DNA tolerated for isothermal recombinase polymerase amplification (RPA) of HIV DNA in whole blood [56]. Other forms of degraded DNA, such as formalin-fixed paraffin-embedded (FFPE) tissue or ancient DNA samples, may benefit from hybridization’s ability to recover fragmented DNA. Hybridization is also compatible with the addition of chaotropic salts, such as guanidinium thiocyanate, which can simultaneously lyse cells and viruses and preserve fragile nucleic acids and may be particularly useful for capture of RNA [40,73].

We have tested our hybridization method across a range of conditions, but have not yet tested it in sample types other than urine. We have also not applied it to capture of longer DNA (>150 bp) or RNA (any length). The results of previous studies (Table 1), however, suggest that hybridization capture is tolerant to a wide variety of complex sample matrices, supporting the possibility that our protocol may improve hybridization performance in other sample types. We have verified robust performance for DNA fragments from 25–150 bp, target concentrations from 0.5–10^4^ copies/mL, and non-target DNA background from 0–10 μg.

One area where our protocol may require modification is for sample types with high protein concentrations, such as plasma or sputum. If the magnetic beads are added prior to denaturation, according to the current protocol, it is likely that as proteins denature, they will adsorb to the bead surface and impact bead behavior. We have observed that beads become more likely to aggregate after heating in urine samples with proteinuria or blood present, resulting in less effective collection of beads on the magnet during wash steps and a tendency for beads to transfer to qPCR during the elution step. We are currently working on strategies, such as the addition of a Proteinase K digestion step, to mitigate bead loss in this situation. Another limitation resulting from the sequence-specific nature of hybridization capture is that it is designed for isolation and detection of specific target sequences, which will be most relevant for pathogen detection. Our hybridization capture method cannot be used to purify total DNA and is thus not appropriate for next-generation sequencing applications. Hybridization capture can, however, be multiplexed and ongoing work in our lab aims to develop a multiplexed system by adding capture probes to target multiple genomic regions. We have demonstrated feasibility of including multiple capture probes, showing capacity for up to 10 probes with only a minor reduction in recovery (S3 Fig). Additional optimization specifically for multiplexed capture may enable higher-scale multiplexing.

## Conclusions

To increase the diagnostic sensitivity of cfDNA detection in urine, we aimed to develop an improved sample preparation method for short DNA fragments. We selected hybridization capture as a DNA extraction technique likely to perform well for short, dilute cfDNA. To enable successful detection of low concentrations of target-specific cfDNA in clinical urine samples, we pushed the limits of hybridization capture to achieve maximum analytical sensitivity and accommodate large-volume samples. Our final protocol improves upon both alternate urine cfDNA extraction methods [28] and previous hybridization capture protocols for sample preparation of targets other than urine cfDNA (Table 1). It reliably detects 5 copies of dsDNA spiked into 10 mL urine (0.5 copies/mL), recovers nearly 100% of short fragments independent of fragment length (25–150 bp), and enables amplification of cfDNA from variable 10 mL urine samples in a single qPCR well (500X concentration). Combining hybridization with an ultrashort qPCR design, we demonstrate detection of DNA fragments as short as 25 bp with high efficiency. Our method meets several design criteria for short urine cfDNA not satisfied by previous urine cfDNA extraction methods and hybridization capture protocols: (1) high recovery of short fragments, (2) large input sample volume, and (3) <1 copy/mL sensitivity. Two key design features ensure its robust performance and unprecedented sensitivity: a two-probe system for recovery of both strands of dsDNA and dual biotinylated capture probes to facilitate optimal probe density, increase thermostability of the bead-probe linkage, and eliminate interference from endogenous biotin. The strong analytical performance of our hybridization capture method described here suggests that the approach will increase recovery of short, dilute urine cfDNA and contribute to increased sensitivity of urine cfDNA-based diagnostic tests. Design of capture probes for new targets is straightforward, and, in our experience, does not require design iterations or assay optimization to achieve high recovery. We believe our hybridization method will be versatile and useful for broad applications in the urine cfDNA field, and may also have utility for other targets and sample types. We hope that the detailed, user-ready protocol and probe design guidelines included here will enable reproduction of our results, transfer of the hybridization assay to new users, and improved sample preparation for wide-ranging applications benefiting from sequence-specific purification.

## Supporting information

S1 Fig Justification for occasional >100% recovery. In some cases, the calculated percent recovery of our hybridization assay may be >100% due to expected variations when quantifying by a qPCR standard curve. The relationship between qPCR threshold cycle and calculated starting quantity is exponential, so a small change in threshold cycle (for either the experimental sample or the qPCR standards used to generate the standard curve) can result in a large change in calculated starting quantity, and therefore percent recovery. Given here are the qPCR amplification curves for three example experiments with 70%, 99%, and 127% calculated recovery. The experimental samples (1000 copies extracted by hybridization) are shown as green curves with hollow circles. The qPCR standards (10, 10^2^, 10^3^, 10^4^, 10^5^ copies) are shown in grey and the NTCs (0 copies) are shown in red. The line at 500 RFU indicates the baseline threshold at which the threshold cycles were determined. Despite the variation in calculated percent recovery caused by minor differences in the standard curves, the 1000 copy hybridization output visually overlaps with the 1000 copy PCR standard across all three experiments, indicating near complete recovery of DNA by hybridization. We run a new qPCR standard curve for every experiment, so while calculated percent recovery may diverge from 100% for technical replicates from an individual experiment, we expect the calculated percent recovery across multiple independent experiments to center around 100%, as seen in Fig 3A. Minor differences in calculated percent recovery for experiments with only technical replicates from the same experiment and/or quantified based on different standard curves are not necessarily meaningful and should be interpreted with this in mind (*e*.*g*., Fig 4C–4E).
(TIF)

S2 Fig Increasing hybridization temperature or time has no effect on recovery. 1000 copies of positive control target were extracted from 10 mL pooled urine with hybridization at room temperature or 45°C for 30 min or 60 min (n = 3 technical replicates from the same experiment).
(TIF)

S3 Fig Preliminary demonstration of multiplexing capability. Without additional assay optimization, multiplexed capture of up to 5 dsDNA targets or 10 ssDNA targets is possible with only moderate reduction in recovery compared to single-plex capture. To represent conditions expected during multiplexed capture, the ratio of target-specific probes to non-target probes was varied (100:0, 50:50, 20:80, 10:90) while keeping the total probe concentration constant (50 pmol per 50 μL beads) (n = 3 technical replicates per condition; 10^3^ copies 50 bp dsDNA input).
(TIF)

S1 Text Recommended designs to ensure specificity when targeting short fragments. **(A)** Primer design and optimization of PCR annealing temperature to encourage specific amplification. **(B)** Addition of spacer region to synthetic positive control to easily identify contamination and prevent false positives during clinical testing.
(PDF)
