# Supplementary figures and images for: Ultrasensitive hybridization capture: Reliable detection of <1 copy/mL short cell-free DNA from large-volume urine samples
Source: PLoS One. 2021 Feb 26;16(2):e0247851. doi: 10.1371/journal.pone.0247851 (PMC7909704; doi:10.1371/journal.pone.0247851)

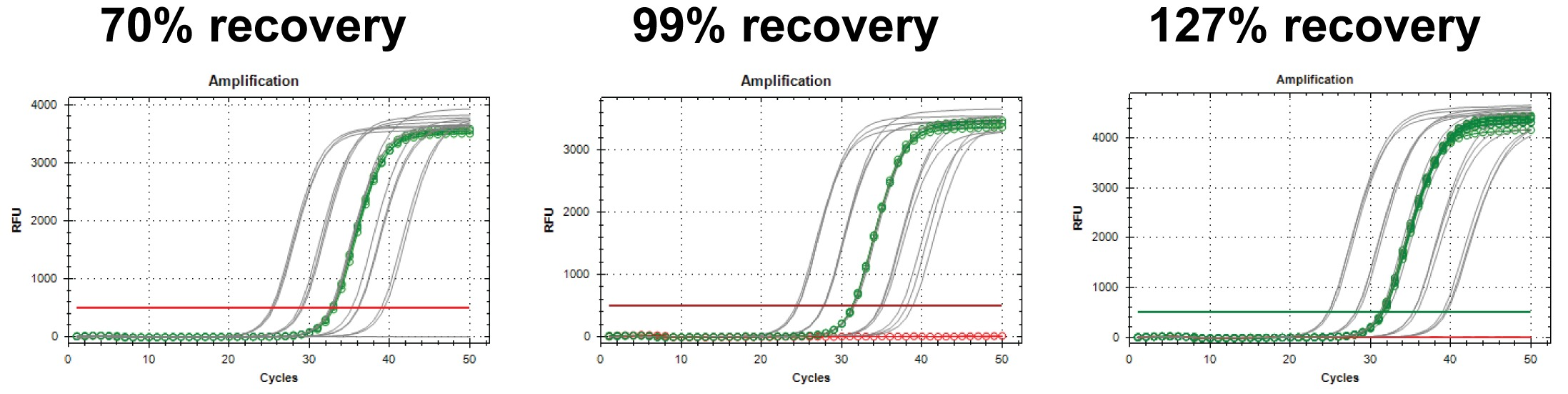

Supplement: S1 Fig — In some cases, the calculated percent recovery of our hybridization assay may be >100% due to expected variations when quantifying by a qPCR standard curve. The relationship between qPCR threshold cycle and calculated starting quantity is exponential, so a small change in threshold cycle (for either the experimental sample or the qPCR standards used to generate the standard curve) can result in a large change in calculated starting quantity, and therefore percent recovery. Given here are the qPCR amplification curves for three example experiments with 70%, 99%, and 127% calculated recovery. The experimental samples (1000 copies extracted by hybridization) are shown as green curves with hollow circles. The qPCR standards (10, 102, 103, 104, 105 copies) are shown in grey and the NTCs (0 copies) are shown in red. The line at 500 RFU indicates the baseline threshold at which the threshold cycles were determined. Despite the variation in calculated percent recovery caused by minor differences in the standard curves, the 1000 copy hybridization output visually overlaps with the 1000 copy PCR standard across all three experiments, indicating near complete recovery of DNA by hybridization. We run a new qPCR standard curve for every experiment, so while calculated percent recovery may diverge from 100% for technical replicates from an individual experiment, we expect the calculated percent recovery across multiple independent experiments to center around 100%, as seen in Fig 3A. Minor differences in calculated percent recovery for experiments with only technical replicates from the same experiment and/or quantified based on different standard curves are not necessarily meaningful and should be interpreted with this in mind (e.g., Fig 4C–4E). (TIF) [file pone.0247851.s001.tif]

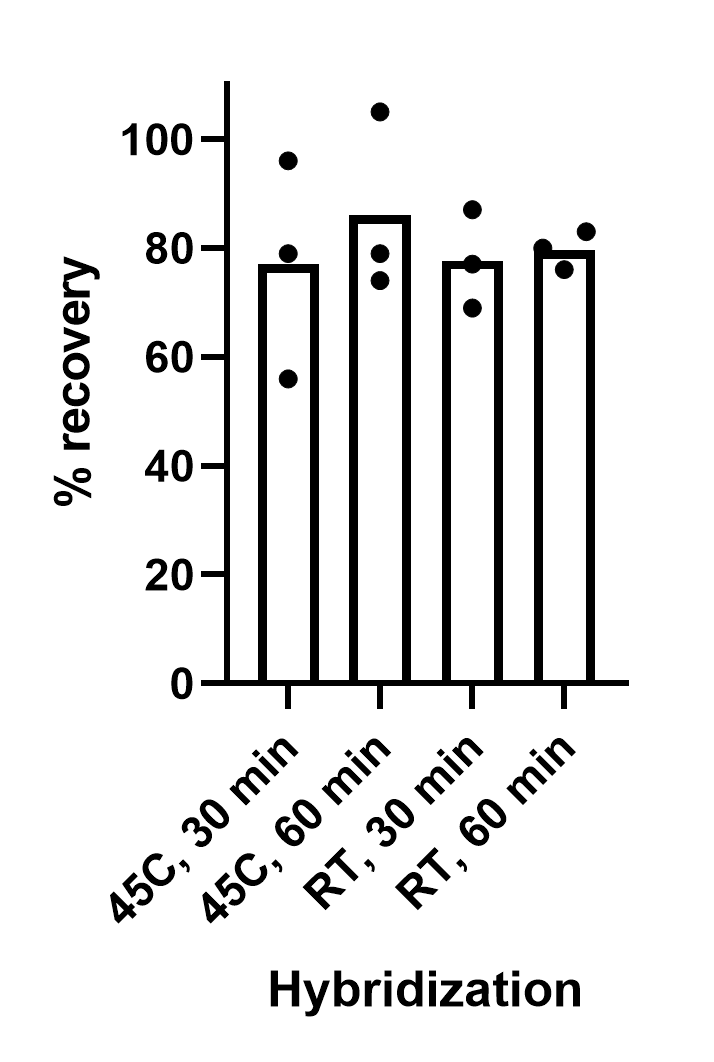

Supplement: S2 Fig — 1000 copies of positive control target were extracted from 10 mL pooled urine with hybridization at room temperature or 45°C for 30 min or 60 min (n = 3 technical replicates from the same experiment). (TIF) [file pone.0247851.s002.tif]

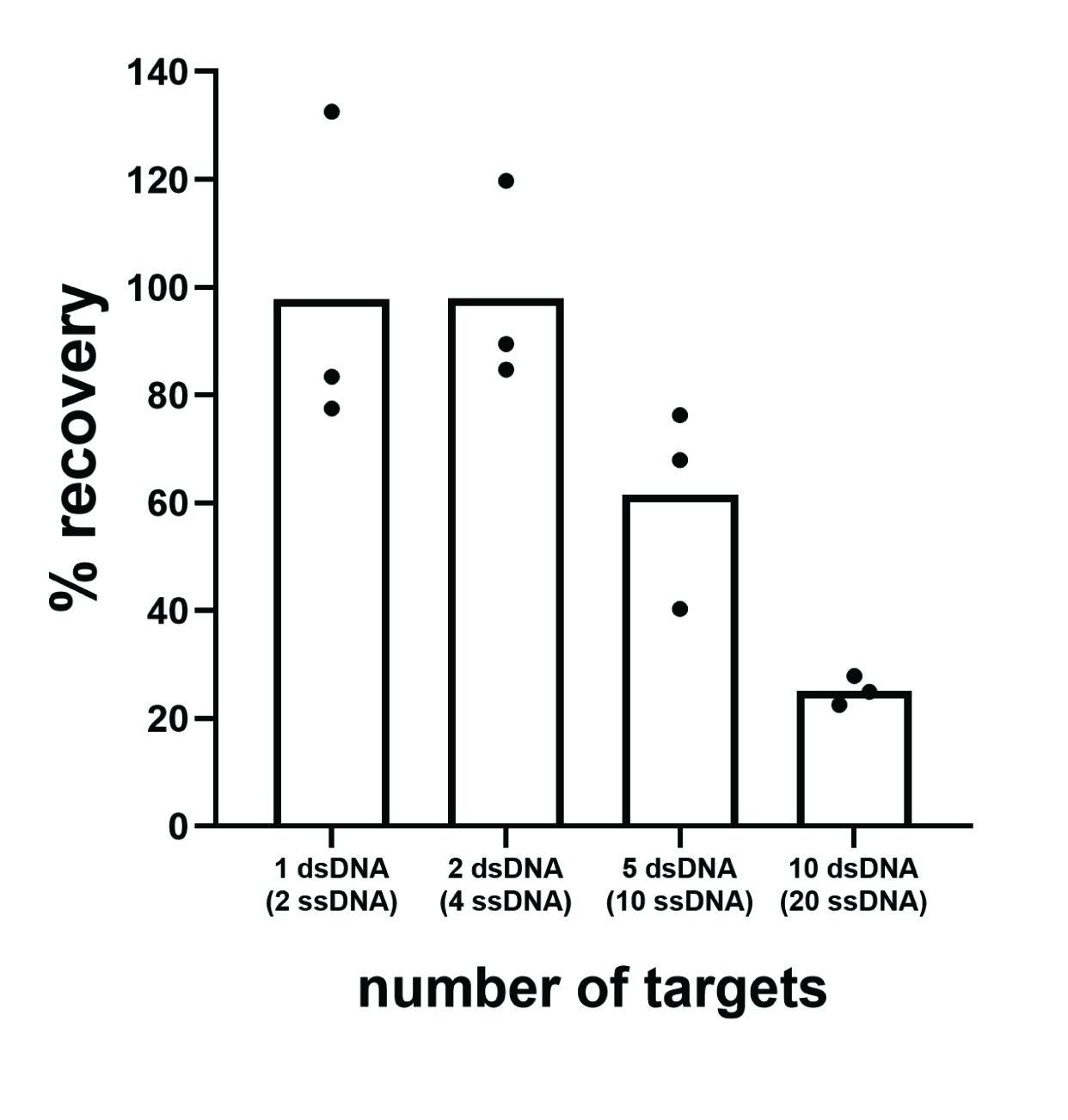

Supplement: S3 Fig — Without additional assay optimization, multiplexed capture of up to 5 dsDNA targets or 10 ssDNA targets is possible with only moderate reduction in recovery compared to single-plex capture. To represent conditions expected during multiplexed capture, the ratio of target-specific probes to non-target probes was varied (100:0, 50:50, 20:80, 10:90) while keeping the total probe concentration constant (50 pmol per 50 μL beads) (n = 3 technical replicates per condition; 103 copies 50 bp dsDNA input). (TIF) [file pone.0247851.s003.tif]
