# Supplementary material for: Ultrasensitive hybridization capture: Reliable detection of <1 copy/mL short cell-free DNA from large-volume urine samples
Source: PLoS One. 2021 Feb 26;16(2):e0247851. doi: 10.1371/journal.pone.0247851 (PMC7909704; doi:10.1371/journal.pone.0247851)
Supplement: S1 Text — (A) Primer design and optimization of PCR annealing temperature to encourage specific amplification. (B) Addition of spacer region to synthetic positive control to easily identify contamination and prevent false positives during clinical testing. (PDF) [file pone.0247851.s004.pdf]

S1 Table A: Primer design and optimization of PCR annealing temperature to encourage specific amplification.

Amplicon length must be minimized when targeting short urine cfDNA, leaving no room for a sequence-specific detection probe. Primer design space may also be limited. To ensure specific PCR amplification, we used LNA substitutions ("indicated by "**+X**") to carefully match primer melting temperatures. We increased the PCR annealing temperature to slightly above the empirically-measured primer T_m, which improved specificity (no amplification of 10 ng human genomic DNA) and had no negative effect on PCR efficiency (>97%). While hybridization capture removes most non-target DNA, minor non-specific adsorption of non-target DNA to the bead surface (independent of capture probes) is possible, so well-designed primers remain essential to avoiding non-specific amplification when detecting very low target concentrations. We were unable to completely eliminate non-specific adsorption by blocking beads with BSA, Denhardt's solution, or sheared salmon sperm DNA.

Primer	Sequence	Calculated T_m (IDT OligoAnalyzer)	Measured T_m	PCR annealing temperature
Forward	5'-CGAACCCTGCCCAGGTCGA-3'	67.8°C	62°C	n/a
Original reverse	5'-GTAGCAGACCTCACCTATGTGT-3'	62.7°C	58.5°C	58°C
LNA-modified reverse	5'-GTA**+G**CAGA**+C**CTCACCTATGTGT-3'	67.3°C	63°C	64°C

S1 Table B: Addition of spacer region to synthetic positive control to easily identify contamination and prevent false positives.

For our positive control oligonucleotide, we added a 10 bp synthetic sequence (in bold) between the primer binding regions, so that the positive control could be distinguished from the native TB amplicon by post-amplification melt analysis. This design allows us to easily identify contamination and prevent false positives during future testing of clinical specimens.

Target	Length	Sequence	Measured T_m
Native TB target	40 bp	5'-CGAACCCTGCCCAGGTCGACACATAGGTGAGGTCTGCTAC-3'	75.5-76°C
Synthetic positive control	50 bp	5'-CGAACCCTGCCCAGGTCGACA**CCATTCAACA**CATAGGTGAGGTCTGCTAC-3'	76.5°C
